# The expression and relationship of VEGF and MVD in type I endometrial cancer

**DOI:** 10.1097/MD.0000000000042945

**Published:** 2025-06-27

**Authors:** Suning Bai, Qi Wu, Liyun Song

**Affiliations:** a Department of Gynecology, Hebei General Hospital, Shijiazhuang, China.

**Keywords:** endometrial cancer, immunohistochemical staining, microvascular density, vascular endothelial growth factor

## Abstract

**Background:**

In recent years, with the improvement of people’s living standards, the incidence rate of endometrial cancer shows a rising and younger trend in the world. Early stage endometrial cancer patients have a good prognosis after surgical treatment, but late stage patients have a poor prognosis. Therefore, if biological indicators related to the occurrence and development of endometrial cancer with high sensitivity and specificity can be found, it will provide clinical reference for predicting the prognosis of endometrial cancer and evaluating treatment effectiveness. Vascular endothelial growth factor (VEGF) is a positive regulator of angiogenesis, while microvascular density (MVD) is a quantitative indicator of angiogenesis. This study investigates the expression of VEGF and MVD in endometrial cancer and normal endometrial tissue, and explores their roles in the formation and development of endometrial cancer.

**Method:**

Immunohistochemical technique (SP method) was used to detect the expression of VEGF and MVD in paraffin sections of 38 cases of endometrial cancer and 20 cases of normal endometrium. Statistical analysis was conducted using statistical software SPSS 17.0.

**Results:**

The positivity of VEGF in the endometrial cancer group was significantly higher than that in the normal endometrial group. The expression level of MVD in the endometrial cancer group was significantly higher than that in the normal endometrial group. In endometrial cancer, the expression of VEGF is positively correlated with MVD (*R* = 0.811, *P* < .001).

**Conclusion:**

The expression levels of VEGF and MVD are significantly increased in endometrial cancer, and both are positively correlated in endometrial cancer. MVD is related to the surgical pathological staging, lymph node metastasis, and depth of muscle wall infiltration of endometrial cancer, indicating that local neovascularization and rich blood supply play an important role in the occurrence and development of endometrial cancer. VEGF is related to the depth of muscle wall infiltration in endometrial cancer, but not to surgical pathological staging and lymph node metastasis. It is considered that other angiogenic factors besides VEGF play a role in regulating angiogenesis during surgical pathological staging and lymph node metastasis of endometrial cancer.

## 1. Introduction

Endometrial cancer is a group of epithelial malignant tumors that occur in the endometrium, with endometrial adenocarcinoma being the most common. The incidence rate of endometrial cancer accounts for 20% to 30% of female reproductive tract malignant tumors. The average age of onset for endometrial cancer is 60 years old, with 75% occurring in women over 50 years old. The specific pathogenesis of endometrial cancer is not yet fully understood. At present, it is widely believed that the incidence of endometrial cancer can be divided into 2 types. Type I endometrial cancer is estrogen dependent, accounting for the vast majority of endometrial cancer. Its pathogenesis may be due to the long-term effect of estrogen and the excessive proliferation of the endometrium without progesterone antagonism, leading to endometrial cancer. Patients with type I endometrial cancer are mostly younger, and their pathological type is endometrioid adenocarcinoma, with a better prognosis. This type of patients often have hypertension, obesity, diabetes and other diseases at the same time. Type II endometrial cancer is non estrogen dependent, and there is no definite relationship between its etiology and estrogen. The pathological types of type II endometrial cancer are often rare, such as clear cell carcinoma, mucinous adenocarcinoma, etc, which often occur in elderly women with relatively thin bodies and have a poor prognosis. The prognosis of endometrial cancer is closely related to various factors, including surgical pathological staging, histological type, tumor grade, depth of myometrial invasion, lymph node metastasis, etc. Therefore, if biological indicators related to the occurrence and development of endometrial cancer can be identified and applied in clinical practice, it will provide new targets for the treatment of endometrial cancer.

The occurrence and development of any tumor cannot be separated from the generation of blood vessels to provide nutritional supply. Vascular endothelial growth factor (VEGF) is a positive regulator of angiogenesis, while microvascular density (MVD) is a quantitative indicator of angiogenesis. This study used immunohistochemistry to detect the expression of VEGF and MVD in endometrial cancer, aiming to explore the role of angiogenesis factors in the formation and development of endometrial cancer, provide new ideas for the treatment and prognosis of endometrial cancer, and provide evidence-based medicine evidence for the use of angiogenesis inhibitors in the treatment of endometrial cancer.

## 2. Materials and methods

Collect 38 endometrial cancer patients who underwent endometrial cancer surgery at the Hebei General Hospital from January 2014 to September 2015, with a minimum age of 32 and a maximum age of 78. The pathological results showed that all patients were endometrial adenocarcinoma, and their endometrial cancer specimens were taken as the experimental group. Collect patients who underwent total hysterectomy for benign lesions such as uterine fibroids, adenomyosis, and ovarian cysts during the same period, and report postoperative endometrial pathological results as specimens of proliferative endometrium. A total of 20 cases, with a minimum age of 45 years and a maximum age of 60 years, were used as the control group. In the endometrial cancer group, according to histological grading, 18 cases were highly differentiated (G1), 15 cases were moderately differentiated (G2), and 5 cases were poorly differentiated (G3). According to the pathological staging criteria of FIGO endometrial cancer surgery in 2009, there were 16 cases in stage I, 14 cases in stage II, and 8 cases in stage III; 5 cases of lymph node metastasis and 33 cases of no lymph node metastasis. According to the infiltration depth of the uterine wall <1/2 muscle layer in 10 cases and ≥1/2 muscle layer in 28 cases. All patients have not received physical therapy, chemotherapy, drug therapy, radiation therapy, or biological therapy before surgery.

## 3. Result determination

VEGF is mainly expressed in the cytoplasm of endometrial cancer cells. If it is positive, it is manifested as brownish yellow fine particles distributed in the cytoplasm of the cells. The distribution of positive cells has obvious heterogeneity, and it can be weakly positive in some endothelial cells and smooth muscle cells of certain blood vessels. The expression of VEGF can be evaluated based on the intensity of cell staining and the percentage of staining positive cells. A score of 0 is given for those without color rendering, 1 is given for those with brown color rendering, 2 is given for those with brown color rendering, and 3 is given for those with brown color rendering. A positive cell count of 0% is rated as 0 points, ≤25% is rated as 1 point, 26% to 50% is rated as 2 points, and >50% is rated as 3 points. The evaluation of the results was jointly conducted by 2 experienced pathologists. The sum of the 2 results in a score of 0 to 2 for immunohistochemical negative (‐), 3 to 4 for positive (+), and 5 to 6 for strongly positive (+ +).

The criteria for determining MVD: Specific CD34 antibodies are used to label the endothelial cells of blood vessels, and the endothelial cells will be stained brown yellow. Any single endothelial cell or string of endothelial cells that can be separated from adjacent tumor cells, connective tissue, or microvessels and can appear brown yellow will be counted as a blood vessel, and blood vessels with a lumen diameter >8 red blood cell diameters will not be counted. The counting of MVD follows the method of WEIDNER et al.^[1]^ Firstly, under low magnification, select the 4 fields with clear endothelial cell staining, good background, and the most dense microvasculature in the infiltrating area of the tumor. Then, count all stained microvasculature within the high magnification field of view, and take the average of the counting results of the 4 fields as the number of microvasculature in the slice.

The pathological tissue sections of the endometrium were divided into 2 groups according to proliferative endometrium and endometrioid adenocarcinoma, namely the normal group and the intrauterine cancer group. Immunohistochemical methods were used to detect the expression of VEGF and MVD in the pathological sections of the endometrium. The specific steps were strictly carried out according to the SP immune method.

## 4. Statistical processing

Statistical analysis was conducted using statistical software SPSS 17.0. Quantitative data were analyzed using *t*/*t*′ test, one-way ANOVA, or non-parametric test. Count data were analyzed using 2-test or Fish exact probability method. Correlation test was performed using Spearman rank correlation analysis. take α = 0.05 is the testing level, and *P* ≤ .05 can be considered statistically significant differences.

## 5. Results

**Figure 1.**
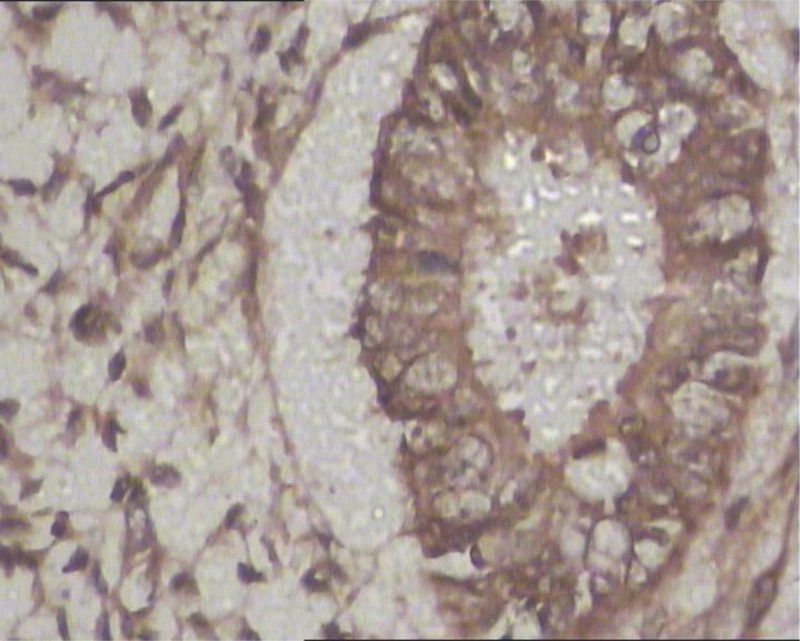
The expression of VEGF in normal endometrium. VEGF = vascular endothelial growth factor.

**Figure 2.**
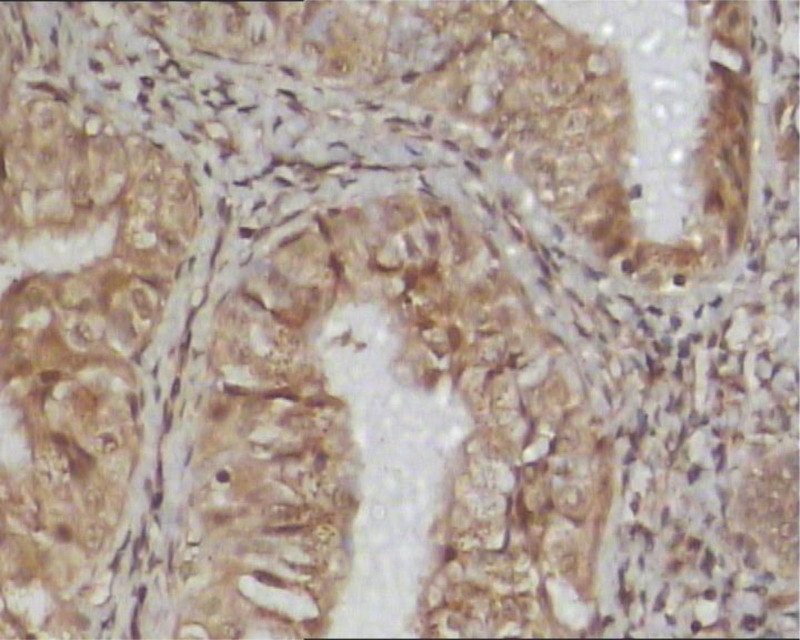
The expression of VEGF in endometrial carcinoma. VEGF = vascular endothelial growth factor.

(1) The positive expression rate of VEGF in the normal endometrial group was 25% (Fig. 1), and in endometrial cancer it was 78.95% (Fig. 2). Compared with the normal endometrial group, the positive expression rate of VEGF in endometrial cancer was significantly higher, and the difference was statistically significant (*χ*^2^ = 15.936, *P* < .001), as shown in Table 1.

**Table 1:** The positive expression rate of VEGF in endometrial carcinoma group and normal endometrium group.

Group	n	VEGF			Positive rate (%)	*χ* ^2^	*P*
		‐	+	+ +			
Normal	20	15	5	0	25	15.936	.00
Endometrial cancer	38	8	16	14	78.95		

VEGF = vascular endothelial growth factor.

In the endometrial cancer group, according to the 2009 FIGO endometrial cancer surgical pathological staging criteria, it can be divided into stage I group, stage II group, and stage III group. The positive expression rate of VEGF in the stage I group is 68.75%, the positive expression rate of VEGF in the stage II group is 78.57%, and the positive expression rate of VEGF in the stage III group is 100%. There is no statistically significant difference in the positive expression rate of VEGF among the 3 groups (*P*_Ⅰ,Ⅱ_ = .689, *P*_Ⅱ,Ⅲ_ = .273, *P*_Ⅰ,Ⅲ_ = .130), as shown in Table 2. According to the depth of cancer cell infiltration into the uterine muscle wall, it can be divided into 2 groups: the depth of muscle wall infiltration ≥ 1/2 group and the depth of muscle wall infiltration <1/2 group. The positive expression rate of VEGF was 89.29% in the group with muscle wall infiltration depth ≥1/2, and 50% in the group with muscle wall infiltration depth <1/2. The difference between the 2 groups was statistically significant (*P* = .019), as shown in Table 3. According to the presence or absence of lymph node metastasis, they are divided into 2 groups: the group with lymph node metastasis and the group without lymph node metastasis. The positive expression rate of VEGF in the group with lymph node metastasis is 100%, and the positive expression rate of VEGF in the group without lymph node metastasis is 75.76%. There is no statistically significant difference between the 2 groups (*P* = .284), as shown in Table 4.

**Table 2:** The positive expression rate of VEGF in different surgical pathologic stages of endometrial carcinoma.

Period	n	VEGF		Positive rate (%)	*P*
		‐	+ ‐ ‐ ‐ + +		
Ⅰ	16	5	11	68.75	*P*_Ⅰ,Ⅱ_ = .689
Ⅱ	14	3	11	78.57	*P*_Ⅱ__,__Ⅲ_ = .273
Ⅲ	8	0	8	100	*P*_Ⅰ__,__Ⅲ_ = .130

VEGF = vascular endothelial growth factor.

**Table 3:** The positive expression rate of VEGF in different muscular wall infiltration depth groups of endometrial carcinoma.

Invasion depth	n	VEGF		Positive rate (%)	*P*
		‐	+ ‐ ‐ ‐ + +		
<1/2	10	5	5	50	.010
≥1/2	28	3	25	89.29	

VEGF = vascular endothelial growth factor.

**Table 4:** The positive expression rate of VEGF in with lymph node metastasis group or no lymph node metastasis group of endometrial carcinoma.

Group	n	VEGF		Positive rate (%)	*P*
		‐	+ ‐ ‐ ‐ + +		
No	33	8	25	75.76	.284
Yes	5	0	5	100	

VEGF = vascular endothelial growth factor.

**Figure 3.**
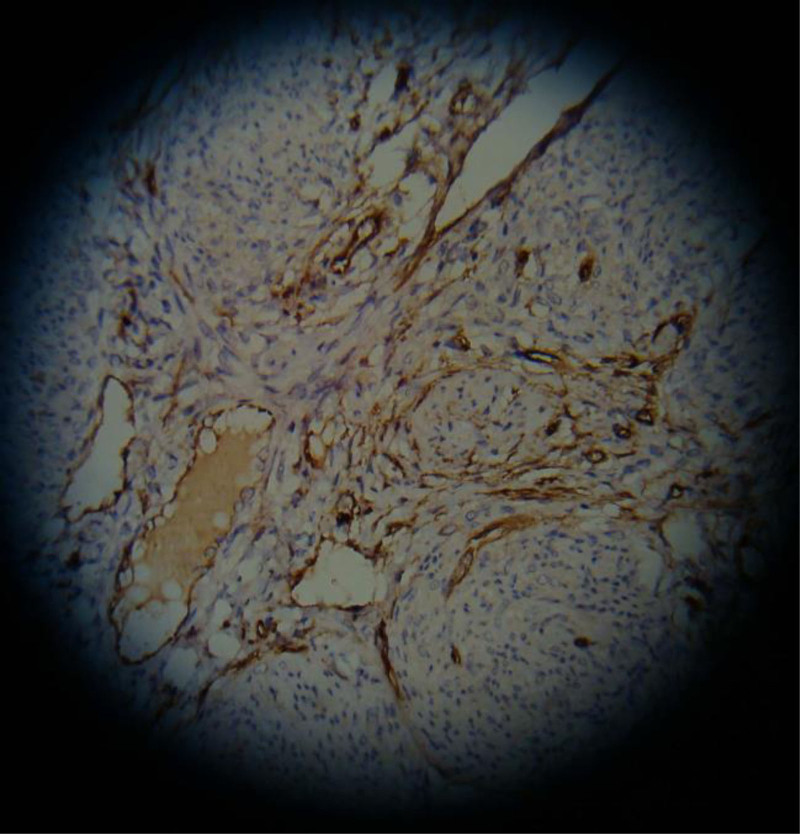
The expression of MVD in normal endometrium. MVD = microvascular density.

**Figure 4.**
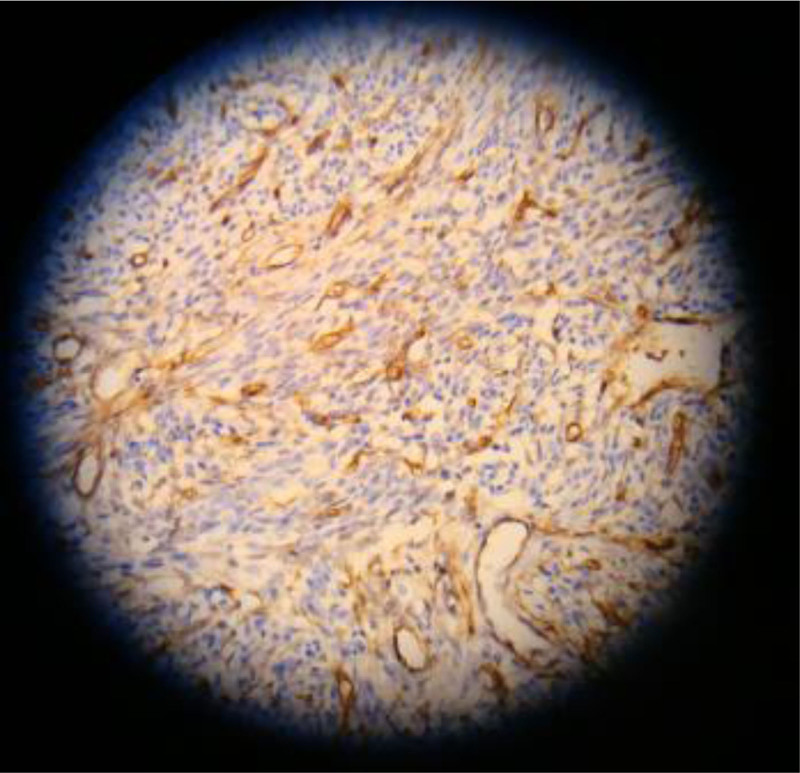
The expression of MVD in endometrial carcinoma. MVD = microvascular density.

(2) The content of MVD in the normal endometrial group was (5.60 ± 0.35) cells/HP (Fig. 3), while in endometrial cancer it was (15.66 ± 1.23) cells/HP (Fig. 4). Compared with the normal endometrial group, the expression level of MVD in endometrial cancer was significantly higher, and the difference was statistically significant (*t*′ = 46.936, *P* < .001), as shown in Table 5.

**Table 5:** The expression of MVD in endometrial carcinoma group and normal endometrium group.

Group	n	MVD (↑/HP)	*t*′	*P*
		($x^{\prime }\pm \;s$)		
Normal	20	5.60 ± 0.35	46.936	.000
Endometrial cancer	38	15.66 ± 1.23		

MVD = microvascular density.

In the endometrial cancer group, the expression level of MVD in the surgical pathological stage I group was (14.73 ± 0.82)/HP, the expression level of MVD in the surgical pathological stage II group was (15.70 ± 0.69)/HP, and the expression level of MVD in the surgical pathological stage III group was (17.44 ± 0.32)/HP. The expression level of MVD in the stage III group was higher than that in the stage II and I groups, and the expression level of MVD in the stage II group was higher than that in the stage I group. The difference in MVD expression levels among the 3 groups was statistically significant (*t*_Ⅰ,Ⅱ_ = 3.442 *P*_Ⅰ,Ⅱ_ = .002, *t*′_Ⅱ,Ⅲ_ = ‐8.006 *P*_Ⅱ,Ⅲ_ < .001, *t*′_Ⅰ,Ⅲ_ = ‐11.553 *P*_Ⅰ,Ⅲ_ < .001); as shown in Table 6. The expression level of MVD was (16.16 ± 0.98)/HP in the group with endometrial cancer muscle wall infiltration depth ≥ 1/2, and (14.25 ± 0.59)/HP in the group with endometrial cancer muscle wall infiltration depth <1/2. The difference between the 2 groups was statistically significant (*t*′ = ‐7.266, *P* < .001), as shown in Table 7. The expression level of MVD in endometrial cancer with lymph node metastasis group was (17.60 ± 0.29)/HP, while in endometrial cancer without lymph node metastasis group, the expression level of MVD was (15.36 ± 1.03)/HP. The difference between the 2 groups was statistically significant (*t*′ = ‐10.159, *P* < .001), as shown in Table 8.

**Table 6:** The expression of MVD in different Surgical pathologic stages of endometrial carcinoma.

Period	n	MVD (↑/HP)	*t*/*t*′	*P*
		($x^{\prime }\pm \;s$)		
Ⅰ	16	14.73 ± 0.82	*t*_ⅠⅡ_ = 3.442	*P*_ⅠⅡ_ = .002
Ⅱ	14	15.70 ± 0.69	*t*′_Ⅱ__Ⅲ_ = ‐8.006	*P*_Ⅱ__Ⅲ_ = .000
Ⅲ	8	17.44 ± 0.32	*t*′_Ⅰ__Ⅲ_ = ‐11.553	*P*_Ⅰ__Ⅲ_ = .000

MVD = microvascular density.

**Table 7:** The expression of MVD in different muscular wall infiltration depth groups of endometrial carcinoma.

Invasion depth	n	MVD (↑/HP)	*t*/*t*′	*P*
		($x^{\prime }\pm \;s$)		
<1/2	10	14.25 ± 0.59	‐7.266	.000
≥1/2	28	16.16 ± 0.98		

MVD = microvascular density.

**Table 8:** The expression of MVD in with lymph node metastasis group or no lymph node metastasis group of endometrial carcinoma.

Lymphatic metastasis	n	MVD (↑/HP)	*t*/*t*′	*P*
		($x^{\prime }\pm \;s$)		
No	33	15.36 ± 1.03	‐10.159	.000
Yes	5	17.60 ± 0.29		

MVD = microvascular density.

(3) There is a significant correlation between the expression of VEGF and MVD in endometrial cancer. After Spearman correlation analysis, it can be concluded that there is a positive correlation between VEGF and MVD expression (*R* = 0.811, *P* < .001).

## 6. Discussion

Endometrial cancer refers to the malignant tumor that occurs in the endometrial epithelium. With the improvement of people’s living standards, its incidence rate and mortality rate show an increasing trend worldwide.^[2]^ And it is the most common cause of death for women.^[3]^ The onset age of endometrial cancer is approximately 65 to 75 years old.^[4]^ The early prognosis of endometrial cancer is good, but the late prognosis is poor.^[5]^ The specific pathogenesis of endometrial cancer is not yet fully understood. Traditionally, it is believed that the types of endometrial cancer can be divided into 2 types. Type I endometrial cancer is estrogen dependent, accounting for the vast majority of endometrial cancer. Patients with type I endometrial cancer are mostly younger, and their pathological type is endometrioid adenocarcinoma, with a better prognosis. Type II endometrial cancer is non estrogen dependent. The pathological type of type II endometrial cancer is often rare, such as clear cell carcinoma, mucinous adenocarcinoma, etc. It often occurs in elderly women with relatively thin body shape, and its prognosis is relatively poor. The prognosis of endometrial cancer is closely related to various factors, including surgical pathological staging, histological type, tumor grade, depth of myometrial invasion, lymph node metastasis, etc. Although the Cancer Genome Atlas Program (TCGA) has improved our understanding of the biological heterogeneity of endometrial cancer, its specific guiding significance for surgical staging, postoperative adjuvant therapy, and follow-up monitoring is limited. Therefore, if biological indicators related to the occurrence and development of endometrial cancer can be identified and applied in clinical practice, it will provide new ideas for the treatment of endometrial cancer.

During the formation and development of any tumor, angiogenesis is necessary to provide the necessary nutrients for its growth. At present, a large number of research results have confirmed that the generation of new blood vessels in tumor tissue is a relatively important factor affecting tumor growth, metastasis, and prognosis. The process of tumor angiogenesis is highly influenced by the host microenvironment and mediators. Therefore, studying the expression of related angiogenic factors, such as vascular endothelial growth factor, at the molecular level in endometrial cancer, and developing targeted therapeutic drugs based on their targets, will provide new ideas for clinical treatment and prognosis monitoring of endometrial cancer.

In recent years, research has found that angiogenesis plays a crucial role in the formation and development of tumors. VEGF is mainly produced by tissue cells and can enhance the ability of small molecule substances to penetrate microvessels and the vascular walls of microvessels. VEGF is a strong pro-angiogenic factor with high specificity, closely related to the growth, invasion, and metastasis of various tumor cells.^[6]^ At present, the vascular endothelial growth factor in the endometrium mainly comes from the glandular epithelial cells and stromal cells of the endometrium, with glandular epithelial cells being the main source. VEGF was initially identified as a multifunctional cytokine in angiogenesis and lymphangiogenesis.^[7]^ In tumor angiogenesis, VEGF promotes the mobilization of inflammatory cells to the tumor site, maintains local inflammatory processes, and induces endothelial cells, platelets, smooth muscle cells, inflammatory cells, fibroblasts, and tumor cells to synthesize pro-angiogenic factors.^[8]^ Tumor associated fibroblasts are the main source of VEGF.^[9]^ Immature cells in the tumor microenvironment secrete more VEGF than mature cells.^[10]^

Studies have found that the expression of VEGF protein in endometrial cancer tissues is significantly higher than that in atypical and proliferative endometrial tissues.^[11]^ The preoperative serum VEGF levels in patients with endometrial cancer are significantly correlated with tumor staging, histological type, grading, uterine myometrial invasion, lymphatic vessel space invasion, and lymph node metastasis.^[12]^ There is research confirming that the positive rate of VEGF and MVD value are relatively high in the lesion tissue of stage II and above type I endometrial cancer patients, indicating that angiogenesis in endometrial tissue may play a crucial role in tumor development and pelvic metastasis.^[6]^ However, there are also studies that have come to the opposite conclusion. Fine et al studied the expression level of VEGF in 47 cases of endometrial malignant tumors and found that VEGF is not related to the metastasis, recurrence, and survival rate of endometrial malignant tumors.^[13]^ Therefore, the significance of VEGF in endometrial cancer needs further research to confirm. At present, the angiogenesis inhibitor Bevacizumab has limited data on its use in the treatment of endometrial cancer. Currently, almost all reports are on the treatment of advanced or recurrent endometrial cancer patients. A retrospective study found that Bevacizumab is an optional palliative treatment for advanced endometrial cancer patients.^[14]^ Therefore, the significance of VEGF in endometrial cancer needs further large-scale research to confirm.

This study found through experiments that the positive expression rate of VEGF in the endometrial cancer group was significantly higher than that in the normal endometrial group, and the difference was statistically significant. Comparing the positive expression rates of VEGF in different stages of endometrial cancer, it was found that there was no significant difference in the positive expression rates of VEGF among stages I, II, and III. The positive expression rate of VEGF was higher in the group with infiltrating muscle wall depth ≥1/2 compared to the group with infiltrating muscle wall depth <1/2, and the difference was statistically significant, indicating that VEGF may play an important role in the biological behavior of endometrial cancer in the occurrence of muscle wall infiltration. There was no significant difference in the positive expression rate of VEGF between the group with lymph node metastasis and the group without lymph node metastasis in endometrial cancer, and the difference was not statistically significant, indicating that VEGF may play a weaker role in the biological behavior of lymph node metastasis in endometrial cancer. The results of this study are inconsistent with some literature reports, possibly due to the small number of cases of lymph node metastasis in this study, which still requires further confirmation through large-scale clinical trials.

MVD can quantitatively reflect the generation of blood vessels in tissues, and it is an indicator that can evaluate angiogenesis. The higher the MVD value, the more abundant the generation of new capillaries. MVD count can serve as a quantitative indicator for analyzing tumor angiogenesis, reflecting the ability of tumor infiltration and metastasis. Currently, immunohistochemical methods are commonly used to label specific molecules on vascular endothelial cells. At present, the most commonly used are CD34, CD105, and factor VIII related antigens, among which CD34 and CD105 are specific glycoproteins of vascular endothelium. This article uses CD34 as a marker molecule and uses immunohistochemistry to detect microvascular density. MVD can reflect angiogenesis. At present, it is widely studied in oncology, mainly including colorectal cancer, renal cancer, breast cancer, bile duct cancer, liver cancer, lung cancer, endometrial cancer, cervical cancer, ovarian cancer and blood related diseases. In addition, research on diseases related to the endometrium, including adenomyosis, endometriosis, endometrial polyps, and endometrial cancer, is also relatively extensive. Wang et al included 29 studies on MVD in endometrial lesions, including 2517 patients. They believed that MVD was associated with deep myometrial invasion, lymph node metastasis, and lower survival rates in endometrial cancer.^[15]^ Another study has found that MVD expression is higher in poorly differentiated endometrial cancers (G2 and G3), and MVD growth coexists with overexpression of VEGF.^[6,16]^ The expression of MVD in endometrial cancer tissue is related to the grading and staging of the tumor. An increase in MVD expression is associated with a decrease in PR expression and an increase in VEGF and Ki-67 expression. Increased MVD is often accompanied by lymph node metastasis.^[17]^

This study found that the expression level of MVD in the endometrial cancer group was significantly higher than that in the normal endometrial group, and the difference was statistically significant. Comparing the expression levels of MVD in endometrial cancer of different stages, it was found that the later the stage, the higher the expression level of MVD, and the difference was statistically significant. The expression level of MVD in the group with infiltrating muscle wall depth ≥1/2 was higher than that in the group with infiltrating muscle wall depth <1/2, and the difference was statistically significant, indicating that microvascular generation plays an important role in the biological behavior of endometrial cancer in the occurrence of muscle wall infiltration, which may provide blood supply and nutritional support. There is a statistically significant difference in the expression of MVD between the group with lymph node metastasis and the group without lymph node metastasis in endometrial cancer, indicating that MVD may also play an important role in the biological behavior of lymph node metastasis in endometrial cancer. This study investigated the expression of VEGF and MVD in endometrial cancer with and without lymph node metastasis and found that microvascularization plays a role in the process of endometrial cancer lymph node metastasis. However, the regulatory mechanism of microvascularization may not be related to the upregulation of MVD expression by VEGF, but may be regulated by other angiogenic factors. Further research is needed to discover the specific factors and mechanisms of action related to it.

The results of this study found a positive correlation between VEGF and MVD expression in endometrial cancer (*R* = 0.811, *P* < .001). The experimental results showed that VEGF positively regulates the generation of blood vessels during the occurrence and development of endometrial cancer, providing nutrients for the occurrence and development of endometrial cancer.

## 7. Conclusion

The expression levels of VEGF and MVD are significantly increased in endometrial cancer, and there is a positive correlation between their expression in endometrial cancer. MVD is related to the surgical pathological staging, lymph node metastasis, and depth of muscle wall infiltration of endometrial cancer, indicating that local neovascularization and rich blood supply play an important role in the occurrence and development of endometrial cancer. VEGF is related to the depth of muscle wall infiltration in endometrial cancer, but not to surgical pathological staging and lymph node metastasis. It is considered that other angiogenic factors besides VEGF play a role in regulating angiogenesis during surgical pathological staging and lymph node metastasis of endometrial cancer. However, the sample size of this study is relatively small, and the research conclusions need further confirmation through large-scale studies.

## Author contributions

**Conceptualization:** Suning Bai.

**Data curation:** Suning Bai, Qi Wu.

**Formal analysis:** Suning Bai.

**Funding acquisition:** Suning Bai.

**Investigation:** Suning Bai.

**Methodology:** Suning Bai, Liyun Song.

**Project administration:** Suning Bai.

**Resources:** Suning Bai.

**Software:** Suning Bai, Qi Wu, Liyun Song.

**Supervision:** Suning Bai.

**Validation:** Suning Bai.

**Visualization:** Suning Bai.

**Writing – original draft:** Suning Bai.

**Writing – review & editing:** Suning Bai, Liyun Song.
